# Scaling Family Voices and Engagement to Measure and Improve Systems Performance and Whole Child Health: Progress and Lessons from the Child and Adolescent Health Measurement Initiative

**DOI:** 10.1007/s10995-023-03755-9

**Published:** 2023-08-25

**Authors:** Christina D. Bethell, Nora Wells, David Bergman, Colleen Reuland, Scott P. Stumbo, Narangerel Gombojav, Lisa A. Simpson

**Affiliations:** 1 Child and Adolescent Health Measurement Initiative, Department of Population, Family and Reproductive Health, Bloomberg School of Public Health, Johns Hopkins University, 615 N. Wolfe Street, Room E4152, Baltimore, MD 21205 USA; 2 Family Voices, 1250 I St NW #250, Washington, DC 20005 USA; 3 Department of Pediatrics, General Pediatrics, Stanford Medicine Children’s Health, MSOB, 1265 Welch Road X240, Palo Alto, CA 94305-5459 USA; 4 Oregon Pediatric Improvement Project, Department of Pediatrics, Division of General Pediatrics, Oregon Health and Sciences University, 707 SW Gaines St, Mail Code CDRC-P, Portland, OR 97239 USA; 5 AcademyHealth, 1666 K St NW #1100, Washington, DC 20006 USA

**Keywords:** Child and adolescent health measurement initiative (CAHMI), Family voices and family engagement, Child health care quality and systems performance measurement, Whole child and family well-being and flourishing, Children’s healthcare system transformation

## Abstract

**Background:**

The 1997 legislation authorizing the United States Child Health Insurance Program sparked progress to measure and publicly report on children’s healthcare services quality and system performance. To meet the moment, the national Child and Adolescent Health Measurement Initiative (CAHMI) public-private collaboration was launched to put families at the center of defining, measuring and using healthcare performance information to drive improved services quality and outcomes.

**Methods:**

Since 1996 the CAHMI followed an intentional path of collaborative action to (1) articulate shared goals for child health and advance a comprehensive, life-course and outcomes-based healthcare performance measurement and reporting framework; (2) collaborate with families, providers, payers and government agencies to specify, validate and support national, state and local use of dozens of framework aligned measures; (3) create novel public-facing digital data query, collection and reporting tools that liberate data findings for use by families, providers, advocates, policymakers, the media and researchers (*Data Resource Center, Well Visit Planner*); and (4) generate field building research and systems change agendas and frameworks (*Prioritizing Possibilities, Engagement In Action*) to catalyze prevention, flourishing and healing centered, trauma-informed, whole child and family engaged approaches, integrated systems and supportive financing and policies.

**Conclusions:**

Lessons call for a restored, sustainable family and community engaged measurement infrastructure, public activation campaigns, and undeterred federal, state and systems leadership that implement policies to incentivize, resource, measure and remove barriers to integrated systems of care that scale family engagement to equitably promote whole child, youth and family well-being. Population health requires effective family engagement.

**Supplementary Information:**

The online version contains supplementary material available at 10.1007/s10995-023-03755-9.

## Significance

Steady progress in maternal and child health policy has led to shifts that increasingly prioritize the engagement of families to shape services and integrate systems of care that equitably promote the early and lifelong health of children. The scope and scale of this progress was shaped, accelerated, and amplified by the quarter century of work of the Child and Adolescent Health Measurement Initiative (CAHMI). Understanding CAHMI’s contributions highlights the fundamental role of measurement based on the voices of families to drive continued systems changes that achieve critically needed improvements required to improve the well-being of all children, youth and families.

**Supplementary Information:**

The online version contains supplementary material available at 10.1007/s10995-023-03755-9.

## Introduction

*“Perhaps herein lies the perspective of the past and the forecast for the future: Society moves forward in terms of what its care, hopes, and aspirations are for its children.” --Katherine B. Oettinger, Fifth Chief of the Children’s Bureau, 1960*.

Healthcare reform efforts between the early 1980s to middle 1990s culminated in new requirements for federal and state agencies and healthcare plans and systems to measure and report on quality and performance (Kaiser Family Foundation, 2011). During this time, healthcare reform focused on reducing healthcare costs using new systems financing models (e.g., managed care organizations) that incentivized reductions in unnecessary services and proactive efforts to address persistent gaps in patient safety and quality of care, including services coordination and preventive care. By the mid-1990s, efforts like the Foundation for Accountability were created to advance the measurement and public reporting of healthcare quality to help public and private sector payers and consumers choose services and share decisions about healthcare, but the focus on child, youth and family health was barely nascent. Measures of children’s healthcare quality or outcomes was only beginning to emerge and largely focused on children with chronic conditions (Newacheck et al., 1996; Pless et al., 2010).

The 1997 passage of legislation authorizing the Child Health Insurance Program (CHIP) sparked the development of the Child and Adolescent Health Measurement Initiative (CAHMI), whose foundational contributions guided and accelerated progress in maternal and child health (MCH). This paper traces the history of CAHMI’s work to create family-centered measures, data, tools and research to inform payers and activate families and drive positive systems change based on the voices of families. Key milestones, lessons and perspectives, as well as instrumental individuals and organizations, are shared to document a key aspect of MCH history and inform continued work to engage families and communities in the measurement and improvement of integrated systems of care focused on the well-being of the whole child and family. Critical policy developments between 1980 and 1997 (Supplementary Table 1) that laid the groundwork for the prioritization of children, youth and families in health care quality measurement, reporting and improvement include:

**1980**

Motivated by soaring health care costs, the Centers for Disease Control and Prevention (CDC) launched the “Healthy People” program to monitor and drive national, state and local disease prevention and health promotion efforts (Lisella & Tate, 1981). The Title V Block Grant was established as a federal-state partnership consolidating several maternal and child health programs (Harwood et al., 2020; Todd and Richards, 2020). The Research Consortium for Children with Chronic Conditions (RCCCC) was also founded to advance knowledge, methods and quality of services (Pless et al., 2010).

**1987**

The US Surgeon General released a call to action for family centered, integrated systems of care for children with special health care needs (CSHCN) (USDHHS, 1987).

**1989**

The Omnibus Budget Reconciliation Act (OBRA) required Medicaid coverage to pregnant women and children age 0–6 (Kaiser Family Foundation, 2011) and (2) strengthened requirements for Title V performance reporting on systems of care for CSHCN (Todd & Richards, 2020).

**1990**

OBRA required Medicaid coverage up to age 18. Healthy People 2020 included systems of care for CSHCN (USDHHS, 1991). The Health Resources and Services Administration (HRSA) launched the national Bright Futures Program to create guidelines for children’s preventive services (Harwood et al., 2020).

**1991**

Family Voices is launched as a coalition of families of CSHCN **(**Wells, 2011**).** The National Committee for Quality Assurance (NCQA) released its first Healthcare Effectiveness Data and Information System (HEDIS) report (Kaiser Family Foundation, 2011).

**1993**

The Government Performance and Results Act was passed, requiring federal agencies to assess and report on performance (Ketti, 1996).

**1994**

The Vermont Child Health Improvement Program established a model leading to the National Improvement Partnership Network to improve children’s healthcare in states (Shaw et al., 2013).

**1995**

The Agency for Healthcare Research and Quality (AHRQ) launched the Consumer Assessment of Healthcare Providers and Systems (CAHPS) program (Kronick, 1996). The Foundation for Accountability began, representing consumers, payers and over 88 million American’s (Skolnick, 1997; McIntyre, 2001).

**1996**

Consumer research led to the FACCT *Consumer Information Framework* as a foundation for the CAHMI framework (Lansky & Bethell, 2001; Hurtado et al., 2001).

**1997**

CHIP was created to expand children’s insurance coverage (Kaiser Family Foundation, 2011). CDC’s Adverse Childhood Experiences (Felitti et al., 1998) and National Academy of Sciences’ “Neurons to Neighborhood” (National Research Council, 2000) studies laid the groundwork to advance neurodevelopmental science and life course health development frameworks making evidence the mandate to transform healthcare to proactively promote the healthy development of children (Garner, 2016). The RCCCC articulates issues to assess quality of managed care plans (Pless, Stein, Walker, 2010).

With foundational funding from the David and Lucile Packard Foundation, the Robert Wood Johnson Foundaton (RWJF) and AHRQ, the CAHMI began as a project of the Foundation for Accountability (FACCT) in 1996 with a planning year to engage families and other stakeholders in specifying goals, principles, methodologic guidelines, a theory of action, priorities and a starting point children’s healthcare quality measurement framework (CAHMI, 2005). FACCT, a small not-for-profit organization operating from 1995 to 2003, had established a strong consortium of public and private sector payers and consumers representing about 88 million Americans (e.g., Medicare/Medicaid, Federal Employees Health Benefits, American Association of Retired Persons and other consumer groups, fortune 500 companies) to create publicly reported healthcare quality information to guide consumer choice and payer decision making. Prioritizing high prevalence and/or high cost adult health condition, there was little focus on children and families. Yet, FACCT research funded by the Center for Medicaid and Medicare Services (CMS) and RWJF led by the founding director of the CAHMI (Christina Bethell) demonstrated children, youth and families were a priority for payers and consumers. At that time, field organizing and advocacy efforts were required to build a coalition and make the case among policymakers and funding agencies that a specific initiative on children’s healthcare quality measurement was required to monitor and improve the ability of the healthcare system to (1) deliver services that promote the healthy development of children and minimize preventable adverse health outcomes; (2) identify and provide effective, appropriate, comprehensive and coordinated treatment for CSHCN and children’s common acute illnesses and injuries; and (3) integrate health, education, social and community services to deliver culturally appropriate, family centered care that also addresses harmful social and family conditions associated with diminished child well-being.

**Fig. 1:**
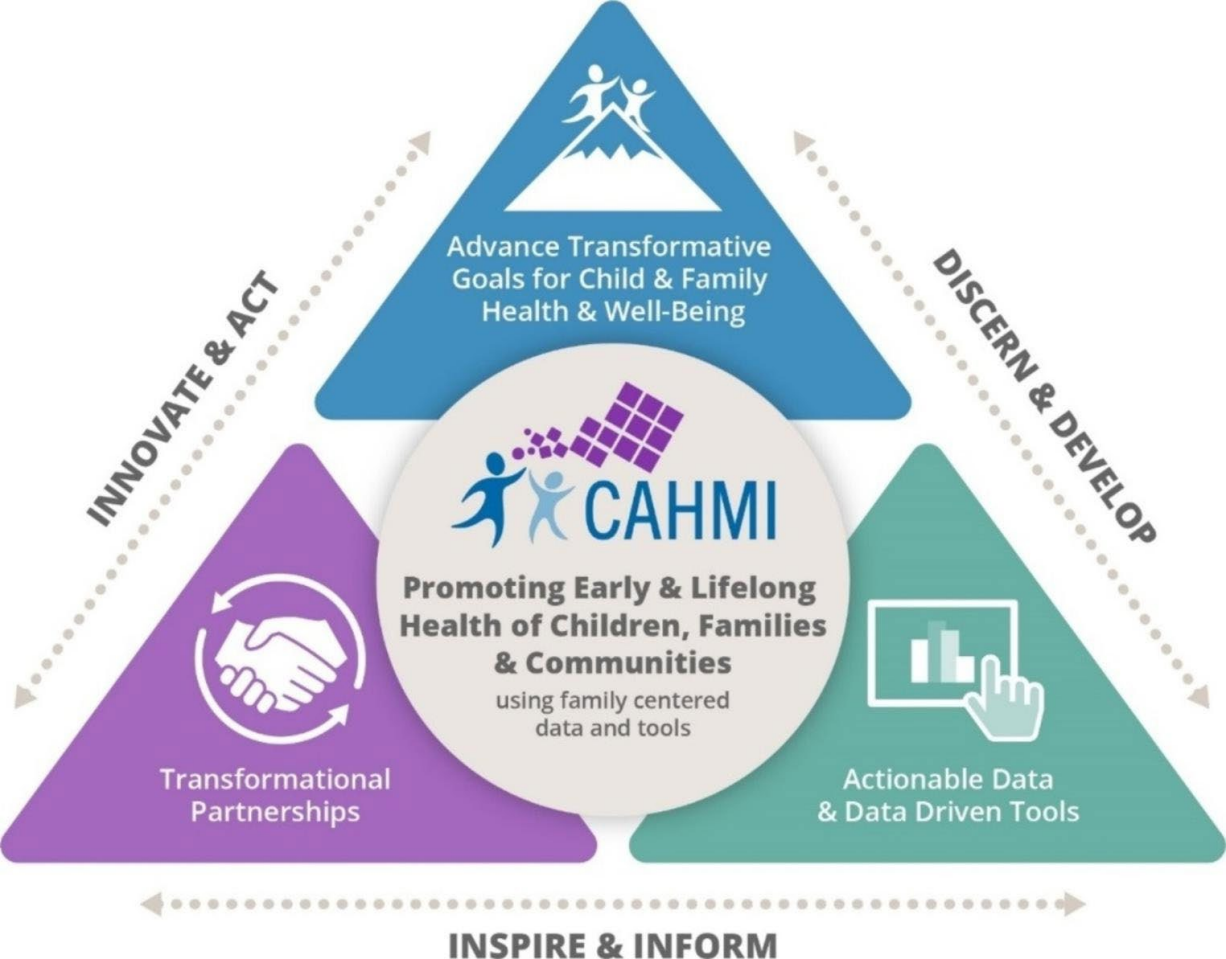
Theory of Action shaping goals and strategies of the Child and Adolescent Health Measurement Initiative (CAHMI)

The CAHMI Advisory Committee (CAHMAC) was formalized in 1997 to establish a quality measurement framework and measurement development process and to review and endorse (or reject) emerging measures. The CAHMAC included Family Voices, federal agencies (CMS, AHRQ, HRSA/MCHB, CDC, Department of Defense (DOD), State Medicaid and Title V program leaders, the American Academy of Pediatrics (AAP), American Board of Pediatrics (ABP), nursing and family medicine associations, NCQA, The Joint Commission (TJC), Children’s Hospital Association (CHA), NICHQ, health plans and other experts (CAHMI, 2002). The work of the CAHMI was guided by deliberate aims to (1) ***keep the focus*** on children, youth and families in healthcare policy, practice and quality improvement; (2) ***build the supply*** of actionable, valid and standardized family-centered quality, systems performance and outcomes measures; and (3) ***build the demand*** and impactful use of family- and youth-reported information to engage families and improve systems performance. As synthesized in Supplementary Tables 1, the CAHMI moved through four distinct phases of work, each of which were anchored to CAHMI’s consistent and explicit theory of action (see Fig. 1), vision, values, objectives, methodologic approaches and strategies (CAHMI, 2005). Each phase of work was met with both eager engagement of family and child focused payers and provider groups as well as with distinct political, professional, and bureaucratic barriers that CAHMI leadership had to overcome. Examples of these are described to ensure an honest recounting of this part of MCH history and to inform future efforts and leaders.

## Phases of Work and Key Milestones

### Phase 1 (1996–2001): Framing and Grounding

“You don’t have to be a genius or a visionary…to be successful. You just need a framework and a dream.” Michael Dell.

CAHMI’s first phase of work (1996–2001) focused on the specification and grounding of a children’s quality measurement framework and new measures into national health plan performance and population-based measurement systems. The CAHMI framework focused on outcomes-oriented domains of quality subsequently included in the initial National Healthcare Quality Report (Kronick, 1996): (1) The Basics (e.g., insurance adequacy, access and customer service); (2) Healthy Development/Staying Healthy (e.g., Bright Futures Guidelines for health promotion and preventive services, HRSA, 2023); (3) Getting Better (e.g., acute care, hospital services); (4) Living With Illness (e.g., care coordination, family support); (5) Changing Needs (e.g., transition and end of life).

**Fig. 2:**
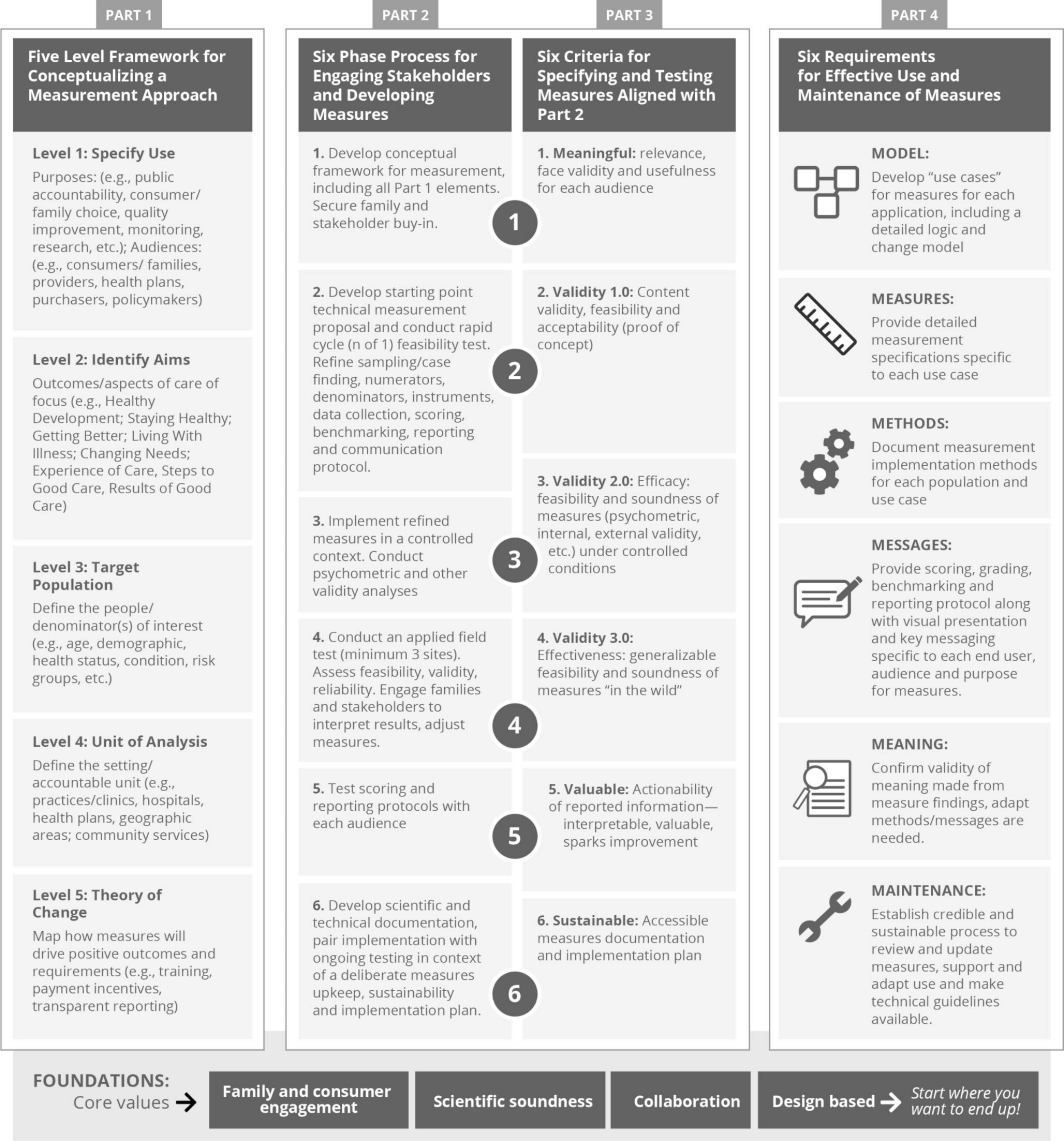
CAHMI’s four-part Measurement In Action design and implementation process^17^

The fact that the framework was led by an organization focused on providing healthcare quality information to payers and consumers and, included, but was *not* led by pediatric providers contributed to initial caution (and at times outright resistance) from some child health care leaders and researchers who were invited to participate and co-lead. Similarly CAHMI was led by a social scientist (Dr. Bethell) and not a pediatrician researcher, which required effective partnership building, an authentic collaborative approach and continued demonstration of credibility to overcome initial skepticism. Early on CAHMI succeeded to build strong stakeholder based advisory and health outcome domain-specific task forces representing family organizations, payers, pediatric organizations and child health research thought leaders (e.g., Ruth Stein, Paul Cleary, Paul Newachek, Neal Halfon, Judith Shaw, James Perrin, Edward Schor, David Bergman, Cynthia Minkovitz, Charlie Homer, Barabara Starfield, Anne Riley) healthcare accrediting bodies, other federal and state agency leaders and funders (CAHMI, 2005). CAHMI convened and facilitated stakeholder tasks forces to specify sets of measures and testing protocols for each domain using the CAHMI’s four-part “Start Where You Want to End Up” measurement development, testing, reporting and endorsement process focused on specific use of measures and relevance to driving health outcomes and improvements in care. See Fig. 2. Measures were collaboratively designed with intensive CAHMI background qualitative, policy and quantitative research and starting point proposals resulting in validated sets of measures generating child/family-centered profiles of performance that integrated information across family and youth-reported experiences of care (e.g., access, caring, communication, shared decision-making), steps to good care (e.g., care standards, patient education, coordination) and results of good care (e.g., quality of life, healthy development, symptom management, school engagement). Design parameters ensured measures could be stratified across child subgroups based on health status, race/ethnicity and other social factors. Extensive analysis of administrative claims and diagnostic data repeatedly resulted in the need for person/family reported measures to create the meaningful, valid, and standardized measures essential to compare performance across health care plans and providers. This emphasis on person/family reported data constituted a major shift in the development of quality measures because the default for pediatric quality measures had been focused on use of administrative data rather than person/family reported data.

Key milestones from 1996 to 2001 are synthesized in Supplementary Table 1. Two stand out. First, the CAHMI framework and education efforts built understanding among healthcare purchasers, families, program leaders and policymakers that “children are not little adults”--children are developing, dependent and disproportionately disadvantaged and require a different approach to healthcare and quality measurement (Forrest et al., 1997). This understanding was inherent in federal and AAP definitions of CSHCN systems of care and Bright Futures Guidelines for preventive and developmental services, which were prioritized for CAHMI measurement development. Secondly, awareness regarding the essential need for family reported measures to standardize measurement and validly compare quality and outcomes grew. Specifically, the CSHCN Screener (Bethell et al., 2015) and CAHPS for Children with Chronic Conditions (CAHPS_CCC) and Medical Home measures were endorsed and included in both NCQA’s Healthcare Effectiveness and Data Information Set (Bethell & Read, 2001; AHRQ, 2012; Bethell & Read, 2002, Bethell et al., 2004b) and were adapted for the new MCHB led National Survey of CSHCN (NS-CSHCN). This contributed to dramatic growth in research and programming for CSHCN (Strickland et al., 2011). Finally, a large national RWJF initiative and state Medicaid programs implemented CAHMI’s CSHCN and preventive care measures- the Young Adults Health Care Survey (YAHCS) and Promoting Healthy Development Survey (PHDS (Bethell & Lansky, 2000; Bethell et al., 2004a, b, c; Bethell et al., 2002; Bethell et al., 2001a, b). The most important factors for success during this phase were the use of a stakeholder-based and payer and child and family centered processes to define the measurement framework and expert, provider and family consensus on methods for developing, validating and reporting on measures. A 2001 national leadership recognition of CAHMI by Family Voices affirmed success in efforts to provide impactful data valued by family advocates (Family Voices, 2021).

### Phase 2 (2002–2008): Data Liberation and Reporting

“You cannot force commitment, what you can do…You nudge a little here, inspire a little there, and provide a role model.” Peter Senge.

CAHMI’s second phase of work (2002–2008) focused on further establishing the validity, reporting and uptake of standardized family centered measures, the creation and launch of the Data Resource Center (DRC-www.childhealthdata.org; CAHMI, 2023a) to provide “point and click” online public access to all national and across state data findings and prepared (“ready to drive”) datasets from the MCHB led National Survey of CSHCN and the forthcoming National Survey of Children’s Health (NSCH), which was designed during this phase of work in close partnership with the CAHMI (Ghandour et al., 2019). Through the HRSA/MCHB sponsored DRC, for the first time, national and across state specific findings on 300 + child health and systems performance measures were easily accessible to all – policymakers, program managers, families, researchers, advocates and the media –-to highlight areas of need, strengths, disparities and variations and inform programs, policies and advocacy. Initiated as an idea in 1999, the DRC online data query and provision of thousands of cleaned and coded datasets and codebooks contributed to hundreds of research papers and the creation of dozens of national and states reports and chartbooks and served as a model and directly supported the creation of other data resources, like RWJF’s The State of Childhood Obesity, America’s Health Rankings, State Health Facts, KidsData.org and Zero to Three’s State of Babies Yearbooks. This use was especially powerful to foster awareness about the need to focus on improving and eliminating socioeconomic inequities in child and youth well-being and facilitated the acceleration of research elucidating associations between child health and their family, neighborhood, education and social context. The CAHMI engaged and trained families, child health leaders, the media and researchers alike to liberate child health data for action using the DRC, bringing to life the DRC’s “Your Data, Your Story” motto and drove policy action.

Supplementary Table 1 synthesizes other milestones reached during this time, including CAHMI’s partnership with MCHB to implement the National Survey of Early Childhood Health (Bethell et al., 2004c) which integrated CAHMI’s PHDS measures, as did numerous state Medicaid programs. The CAHMI launched an online option for families and providers to collect and receive quality of care reports on the PHDS (www.onlinephds.org). New measures of developmental screening and shared decision making were both integrated into the NSCH (Bethell et al., 2011a). New hospital quality measures also emerged (Bethell et al., 2006). Numerous CAHMI led measures were endorsed for use by the National Quality Forum (CAHMI, 2011). Field building work continued to keep the focus on family engagement in national forums typically focused on adult healthcare only (Bethell, 2004).

### Phase 3 (2009–2014): Innovation and Reconciliation

Led by a new paradigm, scientists adopt new instruments and look in new places.

Thomas Kuhn

In Phase 3 (2009–2014) the CAHMI took stock of progress to inform and activate families and improvements in healthcare quality. While tremendous progress occurred to flood the system with family reported data on child, youth and family health and systems performance, progress to use data findings to directly engage, activate and partner with families or to transform and improve healthcare quality or systems was limited by lack of funding. Measurement scoring and reporting on measures also veered from initial recommendations, masking inequities and persistent gaps in quality and outcomes. Also, despite years of available national, across state data health systems and health plan performance thanks to MCHB and NCQA, with some exceptions little to no improvements were observed on key indicators of healthcare system performance (Bethell et al., 2011a, b; Bethell, 2013). Yet, significant variations and pockets of progress did exist across states and for subgroups of children, on measures of medical home for CSHCN (Bethell et al., 2014a, b), developmental screening (Bethell et al., 2011a), childhood obesity (Bethell et al., 2009), school engagement (Bethell et al., 2012), as well as on ACEs and other social and relational determinants of health (Bethell et al., 2014b). The resistance of Medicaid and health plans to implement and optimize use of family reported measures was also a setback, despite evidence and widespread recognition of the inherent limits of healthcare claims, administrative or medical chart data and the value and validity of patient reported quality and outcomes measures. Kulthau et al., (2014) also documents limited progress in advancing more robust children’s healthcare quality measures despite numerous efforts to improve measures and promote their use.

Reconciling these realities, CAHMI pivoted from its primary focus to drive use of quality measures in healthcare to accelerating work to liberate data for advocacy, research and policy, with a focus on family and program leaders and new efforts to compel whole child, integrated systems approaches to healthcare (CAHMI, 2011; Stussman et al., 2013; Bethell et al., 2012). CAHMI lifted up the Oregon Pediatric Improvement Partnership as a stand-alone, new entity as a part of this transition (OPIP, 2023). New research was conducted to feature opportunities for quality improvement using family facing digital tools aimed at directly engaging and activating families to ensure they receive high quality, personalized services based on their goals, needs and context as recommended through Bright Futures Guidelines (e.g., the Well Visit Planner®) (Bethell & Reuland, 2012; Bethell, 2013; Coker et al., 2016).

The WVP operationalizes Bright Futures Guidelines and has been used in a wide range of venues and advanced as an important resource by federal programs (Administration for Children and Families, 2022). It is shown to improve both provider and family experience with WCC visits, dramatically increase screening rates and quality of care and decrease urgent care visits. On a more fundamental level, the WVP provides a foundation for transforming the well child visit through utilization of the time before and between visits to engage and activate families and unburden providers from having to focus on information acquisition, making time for personalized encounters that focus on relationship building and the strengths, needs and priorities of families and linkages to community supports.

Supplementary Table 1 synthesizes these and other milestones from this time, including CAHMI’s co-leadership of the new Maternal and Child Health Measurement Research Network (MCH MRN), creation of an online, searchable MCH Measurement Compendium (CAHMI, 2023b), work with MCHB to transform the NSCH and Title V Block Grant performance measures (Kogan et al., 2015; Ghandour et al., 2018), and continued validation of social, relational and positive health measures (ACEs, child flourishing, early work on school readiness). CAHMI began strategic dissemination of the long awaited, new ACEs data findings from the NSCH through research and advocacy and, with AcademyHealth, launched the *Prioritizing Possibilities* national agenda and research consortium to address ACEs, including over 500 collaborators (Bethell et al., 2017a, b, c). A 2012 national recognition for leadership in MCH boosted commitment to continued work, despite setbacks (Jones et al., 2014).

### Phase 4 (2015-Present): Integration and Transformation

“Without continued growth and progress, such words as improvement, achievement and success have no meaning.” Benjamin Franklin.

**Fig. 3:**
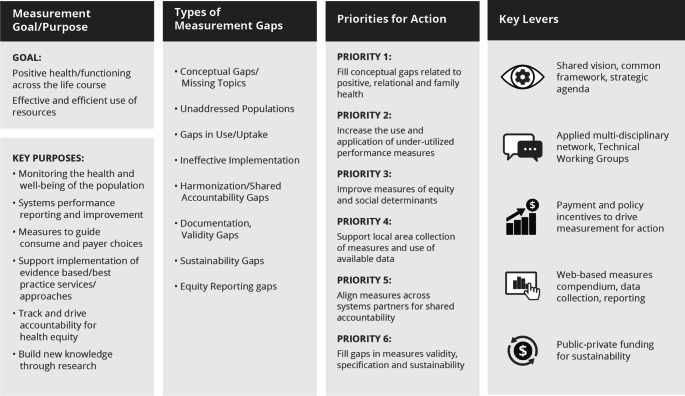
CAHMI led Maternal and Child Health Measurement Research Network Strategic Measurement Agenda Summary (2018)^49^

The most recent phase of the CAHMI (2015 to present) leveraged the accumulated availability of data from new measures to publish collaborative research aimed at catalyzing whole child and family approaches to healthcare policy and practice. During 2016–2020 the CAHMI led the HRSA sponsored Maternal and Child Health Measurement Research Network (MCH MRN) including the online MCH Measurement Compendium and a new national MCH strategic measurement agenda. See Fig. 3. The MCH MRN also led to new family health measures (Weiss-Laxer et al., 2020) and the advancment of measures and research demonstrating distinct impacts of social determinants and relational health factors on children’s physical and mental health, school readiness and engagement and flourishing (Bethell et al., 2017, 2021, 2022; Hirai et al., 2018; Moore et al., 2017). Specification of new Family Resilience and Connection (FRCI) (Bethell et al., 2019) and Positive Childhood Experiences (PCEs) (Bethell et al., 2019) measures led to population-based research showing mitigating impacts on child flourishing and adult mental and relational health even amid the presence of ACEs. This research contributed to the 2021 AAP policy statement on childhood trauma that shifted the focus from reducing toxic stress to promoting relational health through pediatric primary care and integrated systems approaches that prevent childhood trauma and promote flourishing (Audage, 2021). Unfortunately, during this time NQF required revalidation of all endorsed measures and despite substantial work, lack of funding to meet NQF criteria and timelines led to de-endorsement of numerous family reported child health care quality measures.

CAHMI coordinated the *HOPE and a New Science of Thriving* summit to advance consensus for positive approach to public health for children (CAHMI, 2016). The national “Prioritizing Possibilities” agenda was published (Bethell et al., 2017a, b, c), along with over two dozen other papers in a CAHMI led 2017 special issue of Academic Pediatrics (Bethell et al., 2017a). Subsequently, a CHA sponsored and stakeholder team developed the “Payment for Progress” strategy (Bethell et al., 2018) and other state level roadmaps to shape funding and policies to drive the prevention and mitigation of impacts of ACEs to promote child and family flourishing (CAHMI, 2019; Bhushan et al., 2020). This included national congressional testimony (US House Committee On Oversight and Reform, 2018) to advance federal policy attention to address ACEs and foster the social and relational roots of well-being. During this time, a new family driven tool to ensure shared care plans for CSHCN are based on the whole child and family context, aspirations and priorities was created (CAHMI, 2023c; Lucile Packard Foundation for Children’s Health, 2021).

During 2020–2023, with support from RWJF, the CAHMI advanced research and a efforts to scale the implementation of the Cycle of Engaement Well Visit Planner approach (CAHMI, 2023d). Related to this, CAHMI partnered with the HRSA sponsored Mississippi Thrive! Child Health and Development Project and facilitated the development of the Engagement in Action (EnAct!) Framework as a pathway to support national, state and local efforts to create a family engaged, whole child and family well-being focused integrated early childhood health system (CAHMI, 2023e). The partnership with Mississippi Thrive! leveraged CAHMI’s data, measurement, research, policy and stakeholder collaboration expertise leading to a framework that is currently moving along an implementation pathway in Mississippi and is being advanced nationally as a model for use by other state and local areas.

### Operational History

Since 1996, CAHMI optimized its productivity and impact through these four phases of work operating across 27 years solely with grant funding supporting an average of 6 full time equivalent staff, 2 full time equivalent student workers and interns and 2 full time equivalent contractors. Acting as a field building effort, the CAHMI lacked core operational funding, which is common when work is done to build funder commitment rather than follow existing funding opportunities. This content required unrelenting dedication across a nearly three decade political landscape that routinely threatened the very existence of the MCH public health infrastructure. Some of the authors of this history (NW,LAS, DB, SS, NG) believe that it is only the tenacity, enduring optimism, tireless work, collaborative spirit and inspiring vision of CAHMI’s director that sustained the CAHMI over the years. CAHMI has had four operational homes: FACCT for 7 years (FACCT closed in 2004), Kaiser Permanente Center for Health Research (2 year transitional home), Oregon Health & Sciences University (9 years), and Johns Hopkins University (9 years). Since university cultures reward large National Institutes of Health funded research grants and not federal and private foundation funding with lower indirect cost allowances, securing basic infrastructure and development support in an academic context to support CAHMI’s applied and highly technical work also required ongoing resilience.

## Discussion and Six Wishes for Maternal and Child Health

The success of the intervention depends on the interior condition of the intervenor.

William O’Brien.

The Children’s Bureau (now MCHB) was initially charged to “…flow knowledge of the conditions surrounding children’s lives, ideas on how to improve these conditions, and plans and programs for action” (Harwood et al., 2020). As early as 1933, MCHB distributed family facing resources to engage families to promote healthy child development (US Department of Labor, 1933; The Children’s Bureau, 1965). In this way, the quarter century of work of the CAHMI has advanced the mission of MCHB, contributing to the availability and use of standardized measures, digital access to data for all, digital tools to engage and empower families, systems transformation strategies and research that specifically lifts the voices and engagement of families to shine a light on needs and possibilities to translate the science of healthy development to improve MCH outcomes. CAHMI persisted under stable leadership and sustained relationships, which was central to staying the course despite setbacks, absence of operational funding and shifting opportunities to keep the focus on child health.

CAHMI’s work began during a time when policymakers and healthcare payers sought to make information about healthcare quality and costs available to consumers and set in place financial incentives and performance data feedback loops to monitor and drive improvements in quality and health outcomes. However, their dominant focus was on adults with private insurance or in the Medicare program. Without CAHMI’s steadfast contributions on extending this focus to families and child health care in employer sponsored insurance and Medicaid, we would have made slower progress. Yet, despite advances, families still lack access to meaningful information or tools to help make choices or ensure services address their goals, needs and priorities, like the *Well Visit Planner* and *CARE_PATH for Kids.* Research using family generated data shows that only 40% of US children age 3–5 are ready for school (Ghandour et al., 2019) or are flourishing (Bethell et al., 2019), only one-third receive developmental screening before age 3 (Hirai et al., 2018), about half receive care in settings that meet criteria for being a family centered Medical Home and nearly half of US children experience complex medical, social or relational health risks with great impacts on their physical, mental and social well-being (Bethell et al., 2021, 2022). Disparities based on race/ethnicity and income persist.

It is said that “history is merely the study of surprises.” In tracing the history of the CAHMI, what is perhaps the most surprising has been the persistent inaction across most states and local healthcare systems to prioritize child healthcare quality measurement, engage families in care and use and integrate family generated data into electronic records and to create the integrated health, education and social services approaches essential to improving child health outcomes. Inaction extends to implementing existing policies, many of which would result in dramatic improvements in child health, several of which are delineated the Engagement In Action Framework (CAHMI, 2023). For example, Sect. 2713 of the Public Health Service Act requires health plans to ensure provision of Bright Futures Guidelines aligned health promotion and preventive services for children and youth (Federal Register, 2020). Yet, there is little evidence that this is being enforced and advocacy organizations often lack knowledge of such policies or access to system performance information needed to advocate for change. Ironically, consensus is stronger than ever and government agencies like CMS and HRSA emphasize the importance of family/person engagement and person generated health data to focus care on the unique needs, context, goals and priorities for each child and family in order to achieve positive health outcomes (Frampton et al., 2017; Schor & Bergman, 2021; Centers for Medicare and Medicaid Services, 2021; Office of the National Coordinator for Health IT). Longstanding CDC, HRSA and Administration for Children and Families efforts are also in place to advance family-centered, integrated systems approaches in states to improve child health, especially in the area of promoting the healthy development of young children (HRSA, 2022).

As we move forward, six wishes for MCH shared by CAHMI’s director (Dr. Bethell) at 2012 MCH awards ceremony (Jones et al., 2014) are offered here: (1) *Free Our Brilliance*—align healthcare financing, payment, performance measures, incentives and information systems with values to promote healthy development and positive health equity for children; (2) *Take On Transparency*—ensure relevant data is readily available to families, providers, policymakers and advocates to create routine feedback loops that activate, track impact and inform continued innovation and improvement; (3) *Become “We” Ninja’s–* Create a foundation of sustainable collaborative relationships with families and across health, education and social systems in partnership with families and communities; (4) *Prioritize Possibilities*-advance a strengths based, positive construct of health recognizing the absence of illness and risk does not equal the presence of well-being and flourishing; (5) *Take On Trauma*-address the syndemic of childhood trauma in society to ensure services prevent and mitigate sources of toxic stress, promote healthy relationships and scale healing innovations for all children, youth, families and communities; and (6) *Brave Being*—set policies and provide supports that build the well-being of caregivers, teachers, healthcare providers and other adults so that they can be the caring presence “Through Any Door” and “In Every Encounter” that is needed to change systems and promote child well-being.

The very mission of the CAHMI to promote the early and lifelong health of children using family centered research, data and tools worked to reduce avoidable medical, educational and social services and costs, and intentionally sought to transform existing structures in healthcare and related systems. Today, this mission is now appearing center stage, in part due to the contributions of the CAHMI. CAHMI has served as a field builder and a link in the chain in what we hope is an ongoing and new beginning of committed work to ensure family voices and the well-being of children and youth are prioritized to drive a transformed healthcare system where we close unacceptable inequities and gaps in health so that all children and families thrive. Taken as a whole, lessons from the CAHMI call for (1) a restored and sustainable public-private sector family and community engaged measurement infrastructure; (2) direct to public campaigns to activate families that capture and use their voices to spur policy and systems transformations needed to improve child and youth health (Hall et al., 2018); and (3) undeterred federal, state and systems leadership that implement policies to incentivize, resource, measure and remove barriers to integrated systems of care that scale family engagement to equitably promote whole child, youth and family well-being. Population health requires effective family engagement.

## Electronic Supplementary Material

Below is the link to the electronic supplementary material.

Supplementary Table 1: The Child and Adolescent Health Measurement Initiative’s Field Building Journey: Timeline, Themes, Milestones.

## Data Availability

References provide availability.
