# Supplementary material for: Scaling Family Voices and Engagement to Measure and Improve Systems Performance and Whole Child Health: Progress and Lessons from the Child and Adolescent Health Measurement Initiative
Source: Matern Child Health J. 2023 Aug 25;29(8):1153–66. doi: 10.1007/s10995-023-03755-9 (PMC12443881; doi:10.1007/s10995-023-03755-9)
Supplement: Supplementary file 1 — Supplementary Table 1: The Child and Adolescent Health Measurement Initiative’s Field Building Journey: Timeline, Themes, Milestones. [file 10995_2023_3755_MOESM1_ESM.pdf]

Supplementary Table 1

The Child and Adolescent Health Measurement Initiative's Field Building Journey: Timeline, Themes, Milestones

Paper Title: Scaling Family Voices and Engagement to Measure and Improve Systems Performance and Whole Child Health: Progress and Lessons from the Child and Adolescent Health Measurement Initiative

Journal: Maternal and Child Health Journal

Authors: Christina D. Bethell, PhD, MBA, MPH[1], Nora Wells, MEd[2], David Bergman, MD[3], Colleen Reuland, MS[4], Scott P, Stumbo, MA[1], Narangerel Gombojav, PhD[1], Lisa Simpson, MB, BCh, MPH, FAAP [5]

[1]Child and Adolescent Health Measurement Initiative, Department of Population, Family and Reproductive Health, Bloomberg School of Public Health, Johns Hopkins University, 615 N. Wolfe Street, Room E4152, Baltimore, Maryland 21205, USA, cbethell@jhu.edu; ngomboj1@jhu.edu; sstumbo1@jhu.edu
[2]Family Voices, 1250 I St NW #250, Washington, DC 20005, USA, nwells@familyvoices.org
[3]Stanford Medicine Children's Health, Department of Pediatrics, General Pediatrics, MSOB-1265 Welch Road X240 Palo Alto, CA 94305-5459, daberg@stanford.edu
[4]Oregon Pediatric Improvement Project, Department of Pediatrics, Division of General Pediatrics, Oregon Health and Sciences University, 707 SW Gaines St, Mail Code CDRC-P, Portland, OR 97239, reulandc@ohsu.edu
[5]AcademyHealth, 1666 K St NW #1100, Washington, DC 20006, USA, lisa.simpson@academyhealth.org

Corresponding author: Christina D. Bethell, PhD, MBA, MPH. 615 N. Wolfe Street, Room E4152, Baltimore, Maryland 20205. cbethell@jhu.edu

RUNNING HEAD: Scaling Family Engagement to Measure and Improve Child Well-Being

Table 1: The Child and Adolescent Health Measurement Initiative's Field Building Journey: Timeline, Themes, Milestones

1980-1997: **Policy breakthroughs that culminated in a call for a children's healthcare quality and accountability measurement initiative**

1980: Healthy People; Title V Block Grant begin;
1987: Surgeon General CSHCN Report;
1989: Omnibus Budget Reconciliation Act (OBRA) calls for CSHCN Systems; Medicaid required ages 0-6;
1990: OBRA required Medicaid ages 7-18; "Healthy People 2020" adds CSHCN Systems, HRSA launches Bright Futures;
1991: National Committee for Quality Assurance's 1st HEDIS report; Family Voices begins;
1993: Govt' Performance and Results Act passed;
1994: Vermont Child Health Improvement Project seeds National Improvement Partnership Network;
1995: AHRQ Consumer Assessment of Health Plans Survey (CAHPS) program begins; Foundation for Accountability begins;
1997: National Healthcare Quality Report design begins (AHRQ); National Initiative for Children's Healthcare Quality begins; Child Health Insurance Program passed. FACCT's Consumer Information Framework endorsed by payers/consumers representing 88 million covered lives, including Medicare, Medicaid, Fortune 100. Adverse Childhood Experiences and "Neurons to Neighborhood" studies begin to advance life course model.[1-16]

1996-1998: **THEME: Frame, Activate, Create. *Children are not little adults*.**

Family engaged research defines children's quality measurement and reporting framework. Stakeholder process defines principles, goals and strategies to advance children's healthcare quality measurement. CAHMI Advisory Committee created as national public-private partnership and endorses the quality measurement framework, measurement development process and new measures. CAHMI led family-provider-expert measurement teams create and validate measures including the Children With Special Health Care Needs (CSHCN) Screener, Medical Home, the CAHPS- Children with Chronic Conditions (CAHPS-CCC) measurement set, both the Promoting Healthy Development Survey (PHDS) and Young Adult Healthcare Survey (YAHCS) measuring Bright Futures Guidelines recommended care; new Ambulatory Care Sensitive Hospitalization (ACSH), end of life, pediatric asthma measures. Measures begin phase 2 validation using standard methods. Socialization of framework clarified the need for child specific measures recognizing children are developing, dependent and disproportionately disadvantaged.[17-31]

1999-2001: **THEME: Ground, Validate, Deploy. *Start where you want to end up.***

CAHMI's framework outcome domains were incorporated into the National Healthcare Quality Report. Newly validated family centered medical home and CSHCN Screener measures and (CAHPS-CCC) are endorsed by NCQA for use for health plan accreditation and are used in national surveys (MEPS, NS-CSHCN) and for Title V performance monitoring. CMS funds CAHMI to create and validate youth and adult versions of the CSHCN Screener. RWJF funds the National Strategic Indicators study and CAHMI leads use of online versions of the Young Adult Healthcare Survey (YAHCS), CSHCN measures and pediatric asthma and end of life measures. A "Sustainable, Integrated, Actionable" Medicaid measurement implementation approach is set forth to "Swamp the System" with standardized, family centered measures and feedback reports at national, state, local and care setting levels. Continued measurement validation. CAHMI partners with HRSA/MCHB and others to the design the National Survey of CSHCN and National Survey of Children's Health (NSCH).[17-31]

2002-2004: **THEME: Report, Spread, Train. *Have denominator, will travel (and...a definition does not a measure make)***

Measures uptake produces new family centered data for dissemination. Standardized CSHCN denominator (CSHCN Screener) and measures anchored to consensus definitions (e.g., Medical Home, Bright Futures Guidelines) promote further spread and use of measures for health plan quality reports, national surveys (NS-CSHCN, NSCH) and state Medicaid. An online "point and click" public access data dissemination platform (CAHMI's Data Resource Center-DRC) is planned, designed and piloted with families, state agency leads and other end users. The PHDS is used in new National Survey of Early Childhood Health. CAHMI leads a State Learning Network, including External Quality Review Organization trainings to support Medicaid implementation of CAHMI measures anchored to the framework. Measurement design and validation continues. Work begins for new aspirational measures for future use. NSCH launched by MCHB with close CAHMI collaboration. Research conducted from new data to inform policy. Policy work grows to promote family centered systems based on now available data.[32-37]

2005-2008: **THEME: Liberate, Catalyze, Leverage. *Data is, as people do.***

CAHMI provides digital access (www.childhealthdata.org) to national and across state child health and systems performance data on hundreds of new measures to activate and spread use of family centered data to improve systems, services and policies. CAHMI secures national (NQF) endorsement of child measures, advances research on methods and data findings, provides data-in-action trainings with Family Voices, offers technical assistance, co-creates national chartbooks, issue briefs to keep the focus on child health. Partnership with AAP to advance Medical Home and CSHCN data to States. Research publications grow using standardized, accessible family centered measures/data. New Shared Decision Making, Developmental Screening measures validated and integrated nationally. School success factors index and hospital quality survey for limited English proficiency children/families created. Propose and design research to operationalize the Cycle of Engagement model for preventive services for young children leading to the Well Visit Planner.[32-37]

Table 1 (con't): The Child and Adolescent Health Measurement Initiative's Field Building Journey: Timeline, Themes, Milestones

2009-2011: **THEME: Innovate, Advocate, Expand. *Systems transformation was always the point.***

Family driven, whole child and family assessment and goal/priority setting digital Well Visit Planner (WVP) tools are created and tested to drive Cycle of Engagement (COE) strategy and promote guideline-based, personalized and integrated pediatric preventive and developmental services. PHDS goes digital to enable automated quality reports for families and providers as partners in improvement. Medicaid Core Measures process considers CAHMI's CAHPS-CCC and Developmental Screening measures. National Health Interview Survey and Complementary and Alternative Medicine data integrated into the DRC. New Adverse Childhood Experiences (ACEs) and Child Flourishing measures added to NSCH. CAHMI leads *Mindfulness In MCH effort* associated with systems change leadership, healing and preventing childhood trauma and fostering provider/team well-being. Led NQF "Child Health Stream", advanced Childhood Obesity focus, partnered in State CHIPRA Demonstrations, facilitated lift of the Oregon Pediatric Improvement Partnership (OPIP) as a stand-alone effort to drive state and local child health and quality improvement. DRC features expanded to drive state and research use of data to foster systems change.[38-55]

2012-2014: **THEME: Consolidate, Reconcile, Reorient. *No payment alignment, no systems transformation.***

CAHMI partnered closely with HRSA/MCHB to consolidate national surveys (NS-CSHCN, NSCH) and revise Title V performance/outcomes measures. Reconciled losses, including failure of Medicaid/CHIP Core Set of measures to require CAHPS-CCC and few states voluntarily assessing quality for CSCHN. Reorient to greater focus on national/state policy commitment to accountability and pay for performance for child health. Produced research on child health and systems performance across states showing variations based on policy. CAHMI's online MCH Measurement Compendium created via MCH Measurement Research Network (MRN) to track/compare measures across MCH programs and promote shared accountability. Continued research/expansion of the WVP. Led research using new child ACEs and flourishing data to inform health policy action and affirm the need to prioritize childhood trauma and promoting positive and relational health. New family resilience measures created for future use in NSCH. Partner with MCHB to assess school readiness using NSCH. Launched "Prioritizing Possibilities" ("We Are the Medicine") national policy/practice agenda consortium on ACEs and flourishing.[38-55]

2015-2018: **THEME: Reframe, Ignite, Translate. *No mindset shift, no payment alignment.***

CAHMI advances a whole child and family health policy through the CAHMI/AcademyHealth led "Prioritizing Possibilities" national agenda, an Academic Pediatrics special journal issue and national "Payment for Progress" collaboration to advance guideline-based, personalized and systems integrated models that promote the social and relational roots of well-being. DRC revamped for yearly NSCH, Title V metrics. CAHMI leads the MCH MRN network for an updated measurement agenda, new positive, relational and family health measures. WVP is validated in Randomized Controlled Trial. NQF de-endorses measures due to gaps in sustainability capacity to revalidate. Continued partnerships advance a "New Science of Thriving" approach. CARE_PATH for Kids tools created to engage families in shared plans of care for CSHCN. CAHMI partners with Campaign for Trauma Informed Policy and Practice to drive policy action and consults across states to integrated positive and adverse childhood experience science into programs, policies, public education and training.[56-70]

2019-2022: **THEME: Culmination, Restoration, Relationships. *No measures, no mindset shift.***

Culminating research enabled by iterative measurement progress is conducted and helps shift the narrative from childhood toxic stress/trauma as the problem to relational health as the solution. Led research to create/validate a cumulative positive childhood experiences metric and family resilience, parent-child connection, child flourishing measures. Provide Congressional testimony and help states create healing-centered, trauma informed care and financing. A new Integrated Child Risk Index is validated for population-based monitoring of complex medical, social and relational health risks to drive integrated systems approaches. WVP scaling efforts continue to shape early childhood systems integrated, family engaged primary care strategies in states and healthcare. Work grows to translate the science of healthy development, trauma healing and promotion of flourishing and relational systems of care into policy and practice using family centered data and tools linked to payment, accreditation, certification, training, performance measurement and public accountability.[56-70]

Six Wishes for MCH:

- **Free Our Brilliance** (align financing with measures of child health equity and flourishing);
- **Take On Transparency** (create measurement feedback loops linked to inspired quality improvement strategies);
- **Become We Ninjas** (build capacity for data driven partnerships focused on systems integration and relational health skills and structures);
- **Prioritize Possibilities** (create an unstoppable habit of hope with a focus on strengths and what is already whole);
- **Take On Trauma** (relentlessly translate the science of positive and adverse experiences into policy and practice);
- **Brave Being** (child wellbeing requires presence and the well-being of the adults and professionals they encounter and systems transformation and integration of the science of healthy development require relational systems of care)[71-19]
